# The gender dimensions of sexual violence against migrant domestic workers in post-2019 Lebanon

**DOI:** 10.3389/fsoc.2022.1091957

**Published:** 2023-01-18

**Authors:** Jasmin Lilian Diab, Banchi Yimer, Tsigereda Birhanu, Ariane Kitoko, Amira Gidey, Francisca Ankrah

**Affiliations:** ^1^School of Arts and Sciences, Institute for Migration Studies, Lebanese American University, Beirut, Lebanon; ^2^Egna Legna Besidet, Beirut, Lebanon

**Keywords:** migration, labor, gender, domestic work, sexual violence, Lebanon

## Abstract

**Introduction:**

In December 2020, the Lebanese Parliament passed the landmark Law 205 against sexual harassment that could see perpetrators spend up to four years in prison and pay fines up to fifty times the minimum wage. The law additionally affords protection to both the victims and any witnesses who testify against the accused. While the law was applauded as a step forward for sexual harassment victims, it excludes an important faction of the community—migrant domestic workers. The law falls short of international standards by addressing sexual harassment solely as a crime and neglecting to complement this law with labor law reforms, monitoring, and civil remedies. This research focuses on the various forms of sexual violence either protected or enabled under the Kafala system. It aims to depict the incessant violations this type of system has produced.

**Methods:**

Qualitative interviews were conducted with 913 migrant domestic workers in Lebanon. A variety of multifaceted, mixed design methods were used to collect information during the write up of this report, all of which are participatory, inclusive and target group sensitive where needed. These methods ensured that the findings were derived from a collective contribution from a wide range of target groups, triangulated and validated, and that gender considerations were integrated into the data collection and analysis methods. Primarily, these methods included: (1) Desk/Policy Review; and (2) in-depth Key Informant Interviews.

**Results:**

Whilst asked about whether or not they had survived at least one incident of sexual harassment during their employment or stay in Lebanon, 68% of respondents informed the study that they had. According to respondents, various forms of sexual harassment included: (1) inappropriate staring or leering in a sexual manner; (2) sexually suggestive comments/jokes/name-calling; (3) intrusive questions about your sex life/physical appearance that were offensive; (4) someone showing his/her private parts/half or fully-naked body offensively; (5) unwelcome touching, hugging, kissing or other inappropriate physical contact; (6) sexually explicit calls or messages; (7) repeated or inappropriate invitations to dates; (8) sexually explicit pictures, posters or other material; (9) actual or attempted rape or sexual assault; (10) video/photo taking of survivors of a sexual nature; (11) requests or pressure for sex or other sexual acts; and/or (12) other forms of sexual harassment. 56.2% of the sample (513 women) insisted that they had experienced at least one of the aforementioned forms of sexual assault, while 11.7% (107 women) confirmed that they had experienced sexual assault, but weren't willing to describe their experiences in detail.

**Discussion:**

The variety in nationality and race across the sample presented important findings pertaining to ill-treatment, fetishization, and violence each group of women faced. In addition to an overall sense of racism experienced by black MDWs, hierarchy within the MDWs' community presents itself in various forms-even at the early stages of recruitment at the agency. Undocumented MDWs are left powerless in terms of reporting sexual abuse and therefore, are at the mercy of the aggressor. Navigating the country's legal, cultural and social landscapes without documentation or a legal residency permit has become increasingly difficult in recent years, as this has laid the foundation for exploitation and abuse in the areas of: (1) paying less than what MDWs deserve; (2) taking advantage of their legal standing to make them work longer hours; (3) threatening to report them to the authorities if they object; and (4) sexual harassment in all forms.

## 1. Introduction

An estimated 250,000 migrant domestic workers (MDWs) work in Lebanon (Amnesty International, 2019). The majority are women from African and South and South East Asian countries, including Ethiopia, the Philippines, Bangladesh, and Sri Lanka (Amnesty International, 2019). They remain outside Lebanon's labor law protections, and their status in the country remains regulated by the Kafala system–a restrictive immigration regime of laws, regulations, and customary practices that essentially ties migrant domestic workers' legal residency to their employer in a form of labor deemed “modern day slavery” (Harvard International Review, 2020). International Non-governmental Organizations (INGOs) such as Human Rights Watch, Amnesty International, and many others have documented for years how the Kafala system grants employers substantial and unmoderated control over migrant domestic workers' lives (Harvard International Review, 2020).

The system, and the overall lack of a monitoring/reporting mechanism has led to an array of abuses, forced confinement, excessive working hours with no rest days or holidays, not paying MDWs their salaries, as well as verbal, emotional, psychological, physical, and sexual abuse/harassment (Amnesty International, 2020). Women (and men) who leave their employers without “permission” risk losing their legal residency in the country and facing detention and/or deportation (Amnesty International, 2020). In the past, exceptions have reportedly only been made for MDWs in extreme cases of abuse, with the burden of proof falling on the worker, typically leaving workers in situations of forced labor (Human Rights Watch, 2020). According to the International Labour Organization (ILO), the vast majority of the MDWs employed in Lebanon are initially recruited through an agency (ILO, 2016a).

Article 7 of the Lebanese Labor Law explicitly excludes MDWs from the standard labor protections granted to the other categories of employees (ILO, 2010). MDWs across the country have additionally been denied the right to freedom of association and banned from union membership under Article 92 of the Labor Law (ILO, 2010). In application, this essentially means having almost no legal safeguards, leaving them at an increased vulnerability to abuse, trafficking, and exploitation (ILO, 2010). Lebanon's decision to forbid MDWs from forming their own union or being a member of one is considered a violation of the International Covenant on Civil and Political Rights (ICCPR) which the Lebanese Government ratified in 1972 and entered into force in 1976 (United Nations Human Rights, 2021). Article 22 of ICCPR stipulates that “[…] everyone shall have the right to freedom of association with others, including the right to form and join trade unions for the protection of his interests” (United Nations Human Rights, 2021).

### 1.1. The Kafala system in Lebanon

The Kafala system primarily involves a sponsor who has the legal responsibility for a MDW during a contracted period, making the MDW's status in the country and overall livelihood dependent upon the sponsor (FNF, 2021). The system encompasses a number of administrative regulations, customary practices, and legal requirements which bind the worker to the recruiter temporarily. Once in Lebanon, the MDW is assigned an employer. A worker may not change employer or break the terms of the contract unless the employer signs a “release waiver”. A MDW's status in the country becomes irregular/illegal if they leave without the consent of their sponsor and the official release from the authorities. As stated previously, Human Rights Watch continues to insist that the Kafala system in Lebanon puts workers at risk of exploitation and abuse (FNF, 2021). For its part, Anti-Slavery International has stated that the system represents one of the major causes of the continued vulnerability of MDWs (Anti-Slavery International, 2014).

Amnesty International (AI) and Human Rights Watch (HRW) have both documented abuses by recruitment agencies. Many MDWs have also expressed that they were subjected to physical and verbal abuse, forced labor and human trafficking by recruitment agencies in both their sending countries and in Lebanon (Anti-Slavery International, 2014). Recruitment agencies have argued that migrant domestic workers are specifically excluded from Lebanon's labor law, and therefore their relationship with their employer is not monitored, and only governed by limited legal consequences. They further stated that the standard unified contract (as per the Lebanese government's September 2020 decision)^1^ violates the principle of freedom of contract–as both parties should be able to decide on the terms of the contract, including whether they should be bound by the minimum wage. According to both international agencies, the Shura Council's decision failed to take into account both workers' rights and the power imbalances between the parties involved. The Lebanese State Shura Council, which was established by Law No. 10434 of 14 June 1975 (Statute of the State Council), is currently the only administrative jurisdiction in Lebanon (see text footnote 1).

### 1.2. The exclusion of migrant domestic labor from law 205

In December 2020, the Lebanese Parliament passed the landmark Law 205 against sexual harassment that could see perpetrators spend up to four years in prison and pay fines up to fifty times the minimum wage. The law additionally affords protection to both the victims and any witnesses who testify against the accused (CeSSRA, 2020). Beyond criminalizing sexual harassment, the law creates a special fund at the Ministry of Social Affairs for the rehabilitation of the victims (CeSSRA, 2020). In addition to pressing criminal charges, employers and organizations can now impose disciplinary measures, and victims of sexual harassment can seek additional compensation for damages caused. And while the law was applauded as a step forward for sexual harassment victims, it excludes an important faction of the community–migrant domestic workers. According to Human Rights Watch (HRW), the law falls short of international standards by addressing sexual harassment solely as a crime and neglecting to complement this law with labor law reforms, monitoring, and civil remedies (Human Rights Watch, 2021). HRW also insists that the law should provide a clear path for civil remedies for women who may not want to access the criminal justice system or wish to do so alongside criminal complaints (Human Rights Watch, 2021).

Deep and structural power imbalances in the country between different societal groups continues to largely contribute to sexual harassment, discriminatory practices, isolation and precarious legal status. The aforementioned not only put forth additional barriers to reporting abuse, but also exclude migrant domestic workers isolated in private homes and tied to their employers through the Kafala (sponsorship) system, as well as Syrian and Palestinian refugee women who either lack legal residency or who do not have the required work permits.

## 2. Methods

### 2.1. Overview

This research focuses on the various forms of sexual violence either protected or enabled under the Kafala system. It aims to depict the horror, oppression, and incessant violations this type of system has produced. The term “sexual violence” is an all-encompassing, non-legal term that refers to crimes such as sexual assault, sexual harassment, rape, and sexual abuse. The World Health Organization defines sexual violence as “[...] any sexual act or attempt to obtain a sexual act by violence or coercion, act to traffic a person, or act directed against a person's sexuality, regardless of the relationship to the victim” (WHO, 2002). According to the WHO, it is widespread, and is considered to be one of the most traumatic, pervasive, and most common human rights violations (ICRC, 2013).

This large-scale research with interviews of nearly a thousand (913) MDWs in Lebanon serves as an unprecedented, direct look into the intimate experiences of these women, and serves as an output that demystifies sexual harassment both inside and outside professional settings. Empirical patterns and emerging themes identified through the testimonies of Filipino, Cameroonian, Ethiopian, Kenyan, Sierra Leonean, and Sudanese women alarmingly reveal that the overwhelming majority of interviewees experienced sexual harassment and abuse in Lebanon across the last decade.^2^ This statistic, coupled with the act of concealing this reality not only diminishes the power to prevent, curb and fight sexual harassment in Lebanon, but also prevents these women's access to justice. The research reveals that through time, survivors of sexual abuse have increasingly internalized their daily battle with their aggressors, and normalized their incapacity to fight ethnocentric abuses amid a general understanding of the “untouchable” status of Lebanese citizens in their homeland. In their views, foreigners, specifically MDWs, are dehumanized; therefore, any suffering or perpetrated abuse precludes true accountability.

### 2.2. Approach

A variety of multifaceted, mixed design methods were used to collect information during the writeup of this report, all of which are participatory, inclusive and target group sensitive where needed. These methods ensured that the findings were derived from a collective contribution from a wide range of target groups, triangulated and validated, and that gender considerations are integrated into the data collection and analysis methods. Primarily, these methods included: (1) Desk/Policy Review; and (2) Key Informant Interviews (remote and in person where necessary). This study ensured data quality through the application of the BOND Principles (Voice and Inclusion, Appropriate, Triangulation, Contribution, and Transparency) and the ALNAP criteria (Accuracy, Representativeness, Relevance, Generalizability, Attribution, and Clarity around contexts and methods).

The women who took part in these open-ended interviews were informed that they would be taking part in a research, and that this information will solely be used for research purposes. They were informed that the interviews would be anonymous and that their identities would remain anonymous throughout the study in light of the sensitivity of the information provided. Interviews were conducted in accordance with best ethical practice in research, particularly with respect to ensuring participants' safety, anonymity, the protection of data, and risk mitigation. This fieldwork additionally adhered to the Humanitarian Principles of humanity, neutrality, impartiality and operational independence, and were conducted in local dialects and native languages to ensure participants were comfortable expressing themselves and no data was lost in translation. The research team conducted interviews with 913 MDWs workers working and established in Lebanon. These women were aged between 18 and 61+ years old.

### 2.3. Demographic characteristics of the sample and limitations of the study

The 913 women interviewed for this study hailed from: (1) Cameroon, (2) Ethiopia, (3) the Philippines, (4) Ghana, (5) Kenya, (6) Nigeria, (7) Sierra Leone and (8) Srilanka. Women from Ethiopia and the Philippines constituted the majority of the sample (86% combined). Moreover, the majority of respondents were aged between 18 and 38 years old (55% of the sample), followed by those aged between 38 and 60 years old (40% of the sample). In the areas of educational standing, the majority of respondents (50%) stated that they had completed high school, with smaller percentages having completed primary school (35%) and the remainder (15%) stating they had completed a college-level education. Respondents largely worked in the Beirut/Greater Beirut area (45%), followed by Mount Lebanon (40%). The research team reached out to an estimated 1,200 women of which 913 agreed to participate.

Geographical areas outside these two districts such as the North, South, Bekaa and Baalbek-Hermel regions were under-represented in this study due to logistical challenges. The overwhelming majority of respondents lived in Lebanon indefinitely (98%) with much smaller percentages of respondents who took part (less than 2%), informing the study that they had returned to their home countries. When respondents did not live with employers, the study found that they typically live in some form of shared accommodation, whether with a partner or other acquaintances. In the areas of language (conversational level), respondents predominantly highlighted that they speak their native language (95%), while 45% confirmed they speak Arabic, French (1%) and English (36.6%)—with these percentages highlighting that a significant number of women spoke more than one of the aforementioned foreign languages. More than 85% of the sample confirmed that they do know what sexual harassment is prior to taking part in this study—though the extent of their knowledge varies across age, nationality and experience.

## 3. Data analysis

### 3.1. Frequency and type of sexual harassment

Whilst asked about whether or not they had survived at least one incident of sexual harassment during their employment or stay in Lebanon, 68% of respondents informed the study that they had. According to respondents, various forms of sexual harassment included: (1) inappropriate staring or leering in a sexual manner; (2) sexually suggestive comments/jokes/name-calling; (3) intrusive questions about your sex life/physical appearance that were offensive; (4) someone showing his/her private parts/half or fully-naked body offensively; (5) unwelcome touching, hugging, kissing or other inappropriate physical contact; (6) sexually explicit calls or messages; (7) repeated or inappropriate invitations to dates; (8) sexually explicit pictures, posters or other material; (9) actual or attempted rape or sexual assault; (10) video/photo-taking of survivors of a sexual nature; (11) requests or pressure for sex or other sexual acts; and/or (12) other forms of sexual harassment. 56.2% of the sample (513 women) insisted that they had experienced at least one of the aforementioned forms of sexual assault, while 11.7% (107 women) confirmed that they had experienced sexual assault, but weren't willing to describe their experiences in detail.

The most frequent form of harassment (reported by the majority of victims) was unwelcome touching, hugging, kissing, or other forms of inappropriate physical contact. Key informant interviews additionally highlight that MDWs are often approached for sex work and/or to be paid for sexual favors. As one informant who was approached by a group of men explains: “[...] they would ask us for sex and they would offer us money when we are walking on the streets. And when we ignored them, they would insult us.” Assumptions that MDWs are approachable for such “sexual favors” have been rampant in recent years. This has been mainly due to the high number of women trafficked and exploited in prostitution across the country and their forced engagement in non-consensual sex work (KAFA, 2022). A testimony from another key informant further cements this reality. She explains, “[...] Sometimes I feel sad, and helpless. One time I went to a shop to buy something, and the man started saying that ‘I will give you money so that you could have sex with me'. He started touching me and I shouted at him to stop and he would not stop. And other people started to insult me. They were defending the aggressor”.

### 3.2. Harassment in the workplace

While perpetrators of sexual harassment varied across a: (1) male employer, (2) female employer, (3) a person living in the same household, and/or (4) an employer's relative/friend, the overwhelming majority of interviewees who spoke in detail about their experiences, confirmed that the male employer carried out abuses in the majority of the cases (70%). Second to the male employer, relatives and/or friends of employers carried out abuses (40%). Smaller percentages (between 25 and 30%) stated that female employers carried out the abuse, or another individual living in the household (likely a family member or relative). Other perpetrators outside the home/place of employment included taxi drivers (65% of victims who spoke openly about their experience), and police officers (15% of the same segment). One informant describes her encounter with a taxi driver from a “reputable” taxi company. She informs the study, “[...] One time I was in a taxi. I was going from Dora to Mansourieh, and then he took a different route although I know all the roads and shortcuts to Mansourieh. So I asked him why he is going this way, and he said I know, I just wanted to talk to you privately. I told him your job is to drop me at my house. Then he said that I'm so beautiful, sexy, that he liked me, then he tried to reach me with his hands, I was sitting in the backseat at the time. I asked him what he was doing, he said ‘I just wanted to touch you.' Then I got angry. I told him to stop the car and drop me off. He did not stop. I threatened him, and told him that I would jump from the car if he didn't stop. Then he stopped and let me leave”.

These types of incidents were found to have taken place predominantly in the MDW's private room (where available), or around other rooms in the house–with respondents insisting that these incidents have taken place more than once in the same location. Often, such incidents have taken place in the bathroom/shower. One informant describes her experience as “disgusting”. She elaborates: “I don't know why he wanted to do that. I was showering and I wanted to put my clothes on. He just opened the door and came in. He removed his pants, and I shouted at him and told him to get out. A second time this happened, I was also in the bathroom. I pushed him out of the way and left at that time”. A majority of victims “fail to recall” exactly when these types of assault/harassment took place, with many respondents giving time frames ranging from 2010 to present.

### 3.3. Actions taken against perpetrators

When asked about their immediate reaction either during or after harassment took place, more than 95% of respondents who discussed their experiences insisted that they had told the perpetrator to stop. Beyond this course of action, much smaller percentages have taken any actions (legally or otherwise) against the perpetrator. Whilst asked if they had taken any action against the perpetrator, including: (1) reporting them to the police; (2) going to their respective consulates or embassies for help; (3) going to migrant workers' groups/associations/religious groups for help; (4) complaining to the employment agency; (5) complaining to the employer's family; (6) complaining to the employer's neighbor/friend; (7) complaining to their own family/friend; or (8) escaping, percentages smaller than 25% among victims had resorted to at least one of the above. Assistance in the areas of access to justice, legal support, or protection under the Kafala system were found to be a “distant reality” and unattainable to most—ultimately, resulting in an ongoing culture of impunity that permits for a protracted situation of violence, harassment, and abuse to continue to take place.

An important finding, that echoes this reality, is that the majority of survivors opt to “escape” the situation they are in rather than resort to taking any legal action or reporting the incident—though, often, escaping a specific situation of sexual violence does not protect migrant domestic women in the long-term, nor address their indefinite vulnerability. Reasons as to why any further course of action was undertaken against the perpetrator include a fear of the perpetrator, a fear of the perpetrator (or their family)'s retaliation, not knowing where to go for help, a lack of trust in the system in general, and a feel of shame discussing the incident/feeling embarrassed to discuss it. Along these lines, when respondents did in fact decide to take it one step further and reach out to law enforcement, and other forms of support, no tangible actions were taken toward the perpetrator. Rather, survivors of such incidents were “advised to be cautious” (65% of survivors), while others reported that their stories were dismissed, not believed, or that they were simply advised to “run away” if they could.

### 3.4. Links between documentation status, working environment, and harassment

The overwhelming majority of victims (75%) were either undocumented or had their documents confiscated at the time of the harassment. The remaining 25% were documented. On the issue of documentation, it is important to highlight that most MDWs become undocumented as a direct result of their employer confiscating their passport, as well as employers refusing to renew their visas. Power dynamics within the place of employment between MDWs and their employers permit for control over MDWs' bodies, mental health and agency. Importantly, respondents who had their documents confiscated, or were undocumented resorted to authorities/reported abuse less. This, generally linked to a fear of being detained, fined, or forcibly returned to their home country. The majority of survivors (55%) reported that they were independent workers (who simultaneously cleaned multiple houses), 35% that they were live-in workers, and the remaining 10% informing the study that they were unemployed at the time of the harassment. Alongside the sexual harassment, these respondents reported that they experienced other forms of violation in the workplace. These included: (1) unpaid wages/overtime, (2) being required to perform tasks outside their contractual obligations, (3) being deprived of rest/time off, (4) being deprived of food, (5) physical violence, (6) psychological harassment (including gas lighting and re-traumatization), (7) being obliged to perform tasks against their will, (8) spatial segregation (including being banned from sharing spaces or common household items). The highest percentage of responses (more than 65%) reported unpaid wages/overtime, and 55% of respondents reported being required to perform tasks outside their contractual obligations. One respondent states: “[...] they never allowed me to go outside once. They kept me indoors and did not allow me to use my phone before 6 pm. They also underpaid me. Every two months I would receive a payment for one month. When I demanded my salary in full and explained to my madam that I had three kids to feed, my madam got angry and started throwing things at me, so I decided to leave”.

### 3.5. Implications on physical and mental health

Respondents highlighted a multitude of negative implications on their mental and physical health as a result of sexual harassment and other forms of violation in the workplace (that often took place simultaneously). Negative implications on their mental health included (but were not limited to): (1) thoughts about self-harm; (2) anxiety; (3) substance abuse; (4) sleep disorders; (5) depression; (6) stress; (7) eating disorders; (8) withdrawal from society/isolation; (9) suicidal thoughts/tendencies and (10) other implications predominantly outlined as an overall sense of “constantly living in fear”. More than 75% of victims highlighted that they worked in a “stressful” environment–particularly when sexual harassment was taking place. Physical health implications included: (1) irregular menstruation; (2) pregnancy; (3) exhaustion; (4) insomnia; (5) sickness; (6) sexually transmitted infection (STI); (7) injury and (8) other forms of physical implications such as eating disorders, weight loss, and intense migraines. The most common physical health implication reported by the sample was exhaustion (75%), followed by 55% who reported various forms of bodily injuries. Open-ended responses from fieldwork outline that these injuries included bruises, cuts, tears to their vaginal areas, bleeding, and in rare cases a more serious injury such as a sprained arm, wrist, or ankle. However, sexual harassment is not perceived as only physical by the sample. One informant/respondent explains: “[...] sexual harassment does not only mean that someone is forcibly getting physical with you. It is also when someone watches you in a certain way or sexual manner, or exposes his naked body or body parts in front of you”.

### 3.6. Overcoming trauma and referrals

Whilst asked about what would assist the women in overcoming trauma they experienced/continue to experience as a result of the sexual harassment they endured (including: accountability of the abuser, compensation for personal damages, mental health support or counseling, group therapy, de-victimization, including domestic workers in sexual harassment law), the majority of victims (at least 65%) insisted that the accountability of the abuser constituted a priority. Other respondents (between 15-20%) informed the study of their own personal coping mechanisms such as “not opening messages from abusive senders”, “distracting themselves with work”, meditation, and prayer. Importantly, despite the aforementioned, the overwhelming majority of victims (85% of them) insisted that they did not wish to be referred to a doctor or mental health expert, with one respondent informing the study that she “had recovered from her trauma”. The low percentage of women who want to seek out help from a medical or mental health professional is directly linked to their overall fear of consequences if they speak out, as well as their fear that a Lebanese professional would side with the Lebanese perpetrator. Moreover, when undocumented/not in possession of their documents, these women remain reluctant to bring attention to themselves as they navigate their daily lives “below the radar”.

## 4. Findings

### 4.1. Intersections across race and violence intensity

The variety in nationality and race across the sample presented important findings pertaining to ill-treatment, fetishization, and violence each group of women faced. In addition to an overall sense of racism experienced by black MDWs (predominantly expressed by Ethiopian and Cameroonian women throughout this study), hierarchy within the MDWs' community presents itself in various forms–even at the early stages of recruitment at the agency. It is widely reported, and the ILO confirms, that Filipino women are typically “more expensive” to recruit than their Ethiopian, Eritrean, Cameroonian, and Srilankan counterparts (ILO, 2016b)–though this is justified by the expenses associated with bringing these women into the country, respondents highlight that they believe it has to do with race and the tendency of employers to treat blackness as property. As one respondent puts it: “[...] the word ‘aabed' in Arabic, which is used to mean a black person, literally translates into slave. This is important. It is important because it gives them permission to treat us as such.” In comparison to experiences of non-black MDWs (which are not independent of sexual harassment at all) black MDWs express that Lebanese men ‘exotify and objectify black women;' hence leading to a dehumanization of black women, and the propagation of more physical violence against them. As one Key Informant notes: “[...] the rape and abduction of African MDWs seems limitless”.

### 4.2. A culture of impunity within and outside the household

MDWs who experienced rape adamantly alerted the female employer, and in many cases, have altered law enforcement officers and their embassies. None of the women interviewed for the purpose of this study outlined that they received the adequate health or support they needed. Inaction from within the household (namely from the female employer whilst notified) is understood by the MDWs as a means of patriarchy, fear of damage to the family reputation, negligence, or even compliance to the male employer's sexual urges. The absence of accountability foretells a cycle of continuous sexual abuse–particularly as it extends to law enforcement and embassy staff. This has not only led to a sense of helplessness among MDWs who have survived sexual assault, but also led to them not reporting incidents entirely. One informant describes her experience as “disappointing”, and elaborates: “[...] I went to the police. I was in Burj Hammoud, and I was raped by a man who also beat me. I had to go to the hospital, and I needed twelve stitches. I had proof and still have medication for it. I went to the police and they told me that they could not do anything. Even yesterday someone hit me. I stopped going to the police afterwards, they will just laugh at me or just won't do anything”.

### 4.3. Documentation status impacts reporting differently

Undocumented MDWs are left powerless in terms of reporting sexual abuse and therefore, found at the mercy of the aggressor. Navigating the country's legal, cultural and social landscapes without documentation or a legal residency permit has become increasingly difficult in recent years, as this has laid the foundation for exploitation and abuse in the areas of: (1) paying less than what MDWs deserve; (2) taking advantage of their legal standing to make them work longer hours; (3) threatening to report them to the authorities if they object; and (4) sexual harassment in all forms (coupled with the threat to report them or have them arrested. Documentation status, though important for the preservation of the “little to no” rights MDWs have, plays a small role in the success of reporting, as well as these women's abilities to adequately access justice and report perpetrators. Documented MDWs who do in fact press charges against aggressors/perpetrators often unwillingly retract their claims out of fear, do not access justice in any form, are turned away by authorities, and are also turned away by their embassies. While this remained a trend across the majority of interviews, regardless of informants' countries of origin, a number of Filipino MDWs specifically revealed that they had refrained from informing their employers about their being subjected to sexual harassment in public transportation during their day-off, because they believed their employers would use this as an excuse not to let them leave the house again/have another day off.

### 4.4. Mental health support is not typically sought out

Despite the overwhelming findings from this study that highlight the mental health strains that MDWs endure as a result of their experiences of sexual harassment and assault, women interviewed for the study did not express that they had resorted to any form of mental health support. Therapy and counseling sessions are viewed by a majority of women as “shameful” and unnecessary. A number of women also highlight their view that therapy and psychologists can only provide “temporary relief” without addressing more structural and societal problems. As a coping mechanism, many survivors of sexual harassment (in all its forms), have resorted to what they refer to as “various forms of spiritual healing” or to addressing and overcoming trauma individually. Different testimonies from women interviewed include journaling, prayer, meditation, and even group sessions with other survivors where possible and permitted by their employer. According to a team member who conducted fieldwork for this study, many of the women expressed that they were “fine” in a reactive tone whilst asked about how they are coping, “[...] as if trauma and anxiety caused by the events were either played down, or disregarded as too painful to remember”.

### 4.5. Sexual harassment and violence is normalized

According to a team member who conducted fieldwork for this study, many of the women interviewed “smiled or laughed back” when asked if they had been subjected to sexual harassment. Informants' responses outlined a general form of indifference or discontent whilst asked about sexual harassment both inside and outside the workplace—with responses often indicating that this is a recurrent circumstance that is expected as part of the work they carry out in Lebanon, and the overall various forms of racism they endure. Daily aggressions and sexual harassment are largely normalized; for many, a MDW in Lebanon cannot escape sexual harassment—and SGBV becomes inherent to the position, its isolated nature, and the “unconventional circumstances” that the job entails. As one informant explains: “[...] all the women I speak to tell the same story. And the stories gradually get worse. When I was at my Mr.'s house, he would beat me for not sleeping with him. He would try to rape me. He would want to sleep with me all the time and when I refused, he would kick me. Also when I go to the bathroom to take a shower, he tries to come in the bathroom. In the end, he did not pay my salary. I am not the first woman to experience this”. Several cases of abduction, kidnapping, rape, inappropriate touching, sexual commentaries, taxi drivers masturbating and or showing their genitals, and countless incidents of the like were part of informants' testimonies.

### 4.6. Aggressors do not have one profile

As depicted by informants' testimonies, aggressors and perpetrators do not fit one profile, nor do they come from one nationality. They hail from different socioeconomic standings, different genders, different countries, and different professions. The majority of perpetrators were described as men, with the majority of these men being taxi/cab drivers, janitors, and delivery men. This group of men is followed by the “man of the house” where these women are employed or another family member such as a son. As described, the aggressor's origin is not exhaustively Lebanese; though the study finds that the overwhelming majority of perpetrators of sexual harassment are alas Lebanese. Aggressors and perpetrators were aged forty and above; with a significant proportion of respondents highlighting that “older men” were the most violent, aggressive, and insistent with their advances. One informant describes her experience with an “older” man: “[...] I was just shocked at how he was treating me. He wanted to keep making any kind of bodily contact with me all the time. Every morning, he said he would give me fifty dollars if I kissed him. He even would buy me ladies' underwear, and I would not accept them. [...] As these advances persisted, I ran away from the house”.

## 5. Policy recommendations

### 5.1. Enact an urgent ministerial draft law to replace Kafala

There remains a need to replace the Kafala system with an employment contractual system that falls directly under the mandate of the country's Ministry of Labor. This measure will require Lebanon's Parliament to amend Article 7 of the country's Labor Code and supplement it with the necessary legal provisions in line with the ILO's Convention on Domestic Workers (2013) (ILO, 2013), and SDGs 3, 5, 8, 10, and 16 (United Nations, 2022). This step will assist in destructuring the “slavery” nature of the Kafala system, and permit domestic workers to change employers while maintaining their legal immigration status valid. This will assist in alleviating the tendency for wage withholding, passport confiscation, manipulation of power by the employer, detention, and deportation.

### 5.2. Develop a code of conduct for placement agencies and their syndicate

There is a need to revisit and amend the 2009 decree on monitoring and regulating private placement agencies (Swiss Agency for Development and Cooperation, European Union, 2013). Along these lines, a regulation of the requirements needed to issue a recruitment agency license, as well as setting up a strict inspection mechanism is pivotal. Furthermore, accountability measures to halt abuses and violations can fall under this decree, and may be framed under a government body that is tasked with carrying these inspections out.

### 5.3. Criminalize all forms of sexual violence in line with international standards

This can only take place through the amendment of articles 503-520 of the Lebanese Criminal Code, as well as comprehensively defining sexual assault as a violation of bodily integrity and sexual autonomy of every individual regardless of their legal status (Swiss Agency for Development and Cooperation, European Union, 2013). Adopting legislation that specifically defines, criminalizes and provides for appropriate punishment for sexual exploitation and harassment is absent. Moreover, there remains a need to additionally adopt specific provisions in the Lebanese Criminal Code and/or other relevant laws regarding any form of harassment, including verbal sexual harassment.

### 5.4. Ensure that laws adhere to international legal frameworks and standards

Adequate access to justice remains one of the most challenging rights to attain within the community. Local legal frameworks not only fail to adequately define and fully criminalize all forms of GBV, but also fail to include MDWs, refugees, and other marginalized groups within the scope and applicability of this law. At this stage, amending Law No. 293/2014 on domestic violence is pivotal in order to ensure that it comprehensively criminalizes all forms of GBV against women (both inside and outside the MDW community), including by providing for the aggravation of punishment for all domestic violence crimes, and ensuring that all acts of rape^3^ are criminalized.

### 5.5. Enhance justice actors' capacity, logistics, and resources

This is important in order to ensure that women can exercise their full right to access justice in GBV cases. Moreover, there remains a need to increase the overall capacity of the justice system to respond rapidly and effectively to the protection needs of GBV victims through: (1) increasing the duration of protection measures issued by the public prosecutor; and (2) establishing specialized courts and appointing specialized judges as the competent authorities in GBV cases—particularly when GBV survivors are members of the MDW community (or other vulnerable groups such as refugees, gender minorities or stateless persons). Enhancing capacities and resources must additionally extend to increasing the number of forensic experts on each case, and providing them with adequate equipment, kits, and documentation standards to ensure the proper examination of GBV victims.

For MDWs specifically, establishing shelters run or fully funded by the government providing primary/free services including legal assistance, counseling, health care services, and psychosocial support to victims, as well as empowerment services to overcome their physical and psychological injuries is necessary. This can take place in cooperation with already-present CSOs, NGOs, and networks run by, or working from within the community. Amending Law No. 293/2014 to ensure that medical expenses for treatment of GBV victims are paid by the government would largely assist survivors from the community who often leave/flee the perpetrator's home with very little resources and financial capacities.

### 5.6. Ensure that the local legal frameworks are effective and consistent

This is particularly important for Law No. 293/2014 on domestic violence. This will ensure that GBV cases can be effectively and consistently brought to trial, and that prosecutors investigate GBV cases/claims diligently even where no formal complaint has been lodged. Reforming this law will further enable more efficient sanctions for GBV crimes, and that the public prosecutor has the authority to proceed in prosecuting GBV cases even where the victim withdraws their complaint. Reforms to this law will further ensure that the public prosecutor can extend the period whereby they can order the exclusion of an alleged perpetrator from the home. Along these lines, revising local policies to ensure effective judicial measures for addressing and preventing GBV are undertaken when it comes to children (in line with international standards and Lebanon's commitment to the Convention on the Rights of the Child).

## 6. Conclusion

Cultural frameworks and religious beliefs are significant factors influencing women's roles and responsibilities within society. Victims are left to believe that the violations of their rights, especially GBV, are “normal,” and are, thus, pressured not to complain about these violations in order to protect their jobs, regardless of the abuse they suffered. All of this, coupled with the culture of impunity surrounding the treatment of MDWs, results in a normalization of violence—and in turn, sexual harassment. When they report rape and sexual assault allegations, MDWs open themselves up to suspicions of engaging in prostitution (article 523 of the Criminal Code) or to charges of having committed an “affront to public decency” (articles 531-534 of the Criminal Code). Such attitudes on the part of law-enforcement officials have a disproportionate and detrimental impact on MDWs, deterring many of them from reporting the crime because they fear prosecution if their complaint does not result in the successful prosecution of the alleged perpetrator. The unjust legal framework governing MDWs' work and residence in Lebanon, coupled with gender-based social norms and dynamics often hinder MDWs' adequate access to justice in Lebanon and the region at large. The normalization of racism, discrimination, sexual harassment, and social pressure against MDWs constitutes a critical obstacle to the realization of their rights in the short and long term. Evidence-based policy intervention at the institutional and grassroots levels and building toward concrete measures for changing habits and practices remains absent from the country's list of priorities, as well as the labor landscape.

## Data availability statement

The raw data supporting the conclusions of this article will be made available by the authors, without undue reservation.

## Ethics statement

Ethical review and approval was not required for the study on human participants in accordance with the local legislation and institutional requirements. The patients/participants provided their written informed consent to participate in this study.

## Author contributions

JD led on data analysis, methodology design, and write-up. BY, TB, AK, AG, and FA oversaw data collection, conducted fieldwork, and contributed to emerging themes extraction and to write-up. All authors contributed to the article and approved the submitted version.

## Footnotes

^1^The standard unified contract, which the Ministry of Labor adopted on September 8, 2020, included new protections for migrant domestic workers, including vital guarantees against forced labor, and was said to have been an important first step toward abolishing the system of abuse of Kafala.

^2^As part of this study 913 MDWs were interviewed in total: 513 experienced sexual harassment (of which 107 did not want to disclose more info), and 293 reported they did not experience any harassment. The interviewers suspect that many of these women (mostly Ethiopian respondents) were reluctant to speak of their experiences with SH because it is a cultural taboo.

^3^Defining rape as a form of sexual assault that is a physical invasion of a sexual nature without consent or under coercive circumstances. Physical invasion includes penetration, however slight, of any part of the body of the victim – or of the rapist by the victim – with a sexual organ, or of the anal or genital opening of the victim with any object or any other part of the body.
